# Expressions of IGFBP-5, cFLIP in cervical intraepithelial neoplasia, cervical carcinoma and their clinical significances: a molecular pathology

**DOI:** 10.1186/1756-9966-28-70

**Published:** 2009-05-28

**Authors:** Xue-Jing Hou, You-Zhong Zhang, Xin Liu, Li-Hua Meng, Yun-Bo Qiao

**Affiliations:** 1 Department of Obstetrics and Gynecology, Qilu Hospital of Shandong University, Jinan 250012, Shandong, PR China

## Abstract

**Background:**

Insulin-like growth factor binding protein (IGFBPs) have been as potential tumor suppressors in the occurrence and development of tumors. Cellular Fas-associated death domain-like interleukin-1β-converting enzyme (FLICE)-like inhibitory protein (cFLIP) contains a death effect domain (DED), which blocks death receptor pathway and inhibits apoptosis.

**Methods:**

We collected normal cervical tissues from 28 subjects, CIN samples from 37 patients, and cervical cancer tissues from 40 patients. In these samples, we then measured the expression levels of IGFBP-5 and cFLIP via RT-PCR and immunohistochemistry, and we detected the presence of high-risk HPV by Hybrid capture II assays in cervical secretions provided by the subjects.

**Results:**

significant differences in the expression of IGFBP-5 protein among the normal, CIN, and CC tissues (P < 0.05). The highest expression of IGFBP-5 protein was found in CIN stage II and III tissues, whereas the expression of IGFBP-5 in CC samples was decreased relative to controls. The expression level was affected by factors such as clinical stage, pathological differentiation, and lymph node metastasis. Relative to the controls, IGFBP-5 mRNA content was higher in the CC group and lower in the CIN group (P < 0.05). No expression of cFLIP protein or mRNA was detected in normal cervical tissues. However, the degree of pathological changes correlated with increasing expression of cFLIP protein and mRNA, and significant differences were therefore detected between groups (P < 0.05). The HPV infection rates in the CIN and CC groups were much higher than in the normal group (P < 0.05).

**Conclusion:**

IGFBP-5 expression is up-regulated in response to progression of CIN and down-regulated in invasive cervical carcinoma. Detection of IGFBP-5 and cFLIP expression levels, may prove particularly useful for diagnosing and differentiating CIN and CC.

## Background

Cervical carcinoma (CC) is a common cancer of the female reproductive system. Recently, however, the incidence of cervical intraepithelial neoplasia (CIN) has been rising. Development of CIN and CC from normal cervical tissue is a gradual process, though the occurrence and development of these diseases are directly associated with persistent human papilloma virus (HPV) infections. There can be a 10- to 20-year latency between HPV infection and development of cervical carcinoma, and only high-risk HPV infections are not sufficient to induce cellular transformation and tumor occurrence.

Insulin growth factor binding protein 5 (IGFBP-5) is a secreted protein that can bind to insulin-like growth factors, and it can regulate cell growth, differentiation, apoptosis, adherence, and movement. IGFBP-5 has also been shown to play an important role in regulating tumor growth. Cellular Fas-associated death domain-like interleukin-1β-converting enzyme (FLICE)-like inhibitory protein (cFLIP) can block the death receptor pathway, which has the effect of inhibiting apoptosis. In the present study, immunohistochemistry and semi-quantitative RT-PCR were applied to measure the expression levels of IGFBP-5 and cFLIP in normal cervical tissues as well as CIN and CC tissues. This analysis allowed us to assess the potential clinical significance of these proteins to diagnose and differentiate CIN and CC.

## Methods

### Research subjects and grouping

Seventy-seven CIN and CC specimens were surgically removed at the Qi Lu Hospital of Shangdong University between July 2006 and April 2007. Patients included in this study were aged 30 to 82, with an average age of 41 years old. Thirty-seven subjects were diagnosed with different stages of CIN, including 11 cases of CIN stage I, 13 cases of CIN stage II, and 13 cases of CIN stage III. Clinical staging of cervical squamous cell carcinomas was performed according to the Federation International of Gynecology and Obstetrics (FIGO). The CC specimens were classified as stage I (26) or stage II (14). The degrees of tumor differentiation were verified by postoperative pathology, and these included 24 cases of well-differentiated CC and 16 cases of moderately or poorly differentiated CC. Twenty-eight normal cervical tissues were collected to serve as controls. All HE staining sections were rechecked and confirmed by pathology experts, and no patients had been given radiotherapy or chemotherapy.

### Reagents and instruments

Primary antibodies used in this study include IGFBP-5 rabbit anti-human polyclonal antibody (Boster Co., Ltd., Wuhan) and cFLIP rabbit anti-human polyclonal antibody (American Neomarker Co.). The DAB kit (Boster Co., Ltd., Wuhan) was used to reveal positive staining. The Olympus IX81 electric research system inverted microscope was used to examine the sections, and the Hybrid Capture II system (American DIGENE Co.) was used to detect high-risk HPV. Reagents used for RNA extraction and RT-PCR include Trizol, DNA marker (TaKaRa Co.), a reverse transcriptase kit, and a PCR kit (PROMEGA Co.).

### Specimen handling

Tissue samples were drawn from all the specimens after a brief period of culture (20 min) and stored in liquid nitrogen. Additionally, parts of each specimen were fixed in 10% neutral formalin and embedded in paraffin wax. Four serial sections (3–4 mm) were cut from each paraffin block. Cervical secretions from the external cervical orifice and cervical cavity were collected by cervical brush, which was kept in a vial containing HPV cell storage solution. The Hybrid capture II assay was directly applied to these samples to detect high-risk HPV DNA.

### Reverse transcription polymerase chain reaction (RT-PCR)

Total RNA was extracted according to the Trizol protocol. To determine the concentration of the RNA, UV absorbance was measured in a spectrophotometer. cDNA was synthesized by reverse transcription of 2 μg of total RNA. PCR amplification of IGFBP-5 and cFLIP was performed in a final volume of 20 μl, with simultaneous amplification of β-actin as an internal reference. The primers were synthesized by Invitrogen Co., Ltd. (Shanghai). The β-actin primer sequences were forward, 5'-GTGGG GCGCC CCAGG CACCA-3' and reverse 5'-GTCCT TAATG TCACG CACGA TTTC-3', which amplified a band of 540 bp. The forward primer sequence for IGFBP-5 was 5'-AATTCAAGGCTCAGA AGCGA-3', while the reverse primer sequence was 5'-GGCAG AAACT CTGCT GTTCC-3'. These primers amplified a 154 bp band. The forward primer sequence for cFLIP was 5'-CGTGC TGTGT ACCTG CCCAAT-3', and the reverse primer sequence was 5'-CACTGAAAGTCCCCGTCAAC-3' [11]. These primers amplified a 226 bp band.

PCR products were analyzed by 1.5% agarose gel electrophoresis, and they were observed and photographed under ultraviolet light. Band intensities were analyzed by the Touching gel imaging system and compared with β-actin to calculate relative expression levels.

### Immunohistochemical method

Tissue samples were stained with two different antibodies via immunohistochemical method according to conventional staining procedures. Negative and positive controls were run synchronously. For the positive control, CIN and CC tissues were replaced by normal cervical tissues, while for the negative control, phosphate buffer substituted for the primary antibody. Paraffin sections were deparaffinized by routine methods, and antigen retrieval was achieved by microwave treatment. After blocking with serum, IGFBP-5 and cFLIP rabbit anti-human polyclonal antibodies were applied at a dilution of 1:50 and incubated overnight at 37°C. The samples were rinsed three times with PBS (pH 7.2) for 5 min each, then incubated with biotin-labeled goat anti-rabbit IgG for 15 min at 37°C, rinsed again, and incubated with horseradish peroxidase-conjugated streptavidin for 30 min at 37°C. Finally, the sections were rinsed, stained with DAB, re-stained by hematoxylin, dehydrated in an ethanol gradient, cleared in xylene, and fixed by neutral balata.

### Immunohistochemical assessment

This semi-quantitative assay was conducted under a high power lens (×400) integrated with staining intensity and the percent of positive cells. The expression of IGFBP-5 and cFLIP proteins in the histocytes was mostly localized to the cytoplasm, which appeared brownish yellow and contained brownish yellow particles. More than 10 representative fields of each section were observed under high power before we evaluated the staining results. We looked for positive staining within the squamous epithelia of the control group, in the CIN focus position of the CIN group, and in the cancer focus of the CC group. We scored for staining intensity (0: no color; 1: light yellow; 2: brownish yellow; 3: chocolate brown) and the percent of positive cells (0: < 5%; 1: 5 to 25%; 2: 26 to 50%; 3: 51 to 75%; 4: > 75%) separately, and the summation of the two gave the final score (-: 0–2; +: 3–4; ++: 5–6; +++: 7) [12].

### Detection of high risk-HPV

Hybrid capture II assay was applied to directly detect high risk-HPV DNA (American DIGENE Co.). Thirteen HPV subtypes (16/18/31/33/35/39/45/51/52/56/58/59/68) can be detected by this method. In this protocol, double-stranded DNA in the specimen is turned into single-stranded DNA, which is then combined with an RNA probe to form a DNA-RNA hybrid. This hybrid was fixed with a specific antibody, which was subsequently combined with an enzyme-conjugated secondary antibody. The samples were then incubated with a substrate that fluoresces upon encountering the enzyme, such that HPV DNA content in the original specimen can be calculated according to the light intensity signal (RLU). These measured RLU values from the specimens were then divided by the RLU value of a positive control (CO). If the ratio (RLU/CO) of a given specimen was between 0.8 and 1.2, the specimen was weakly positive, whereas less than 0.8 indicated that the specimen was negative.

### Statistical analysis

All of the data were processed by the statistical software package SPSS10.0 and represented as mean ± standard deviation (SD). Kruskal-Wallis test for group comparisons, as well as the Mann-Whitney U test for nonparametric independent two-group comparisons were performed. Differences with P < 0.05 were regarded as statistically significant, P < 0.01 as highly statistically significant.

## Results

### High-risk HPV infection rates

The infection rates of the 13 HPV subtypes in the CIN and CC groups were all significantly higher than in the control group (P < 0.05), while there was no significant difference in the HPV infection rates between the CIN and SCC groups (P > 0.05) (Table 1).

**Table 1:** Infection rate of normal tissue, CIN and Squamous Cell Carcinoma

Group	n	+	-	Infection Rate(%)
Normal tissue	28	6	22	21.4
CIN	37	30	7	81.1*
Squamous Cell Carcinoma	40	36	4	90.0*

*P < 0.05 vs. control

### Expression of IGFBP-5 and cFLIP proteins

The positive staining rate of IGFBP-5 was 71.4% in normal cervical tissues, 91.9% in CIN samples, and 45.0% in CC samples. The expression level in the CIN group was significantly different from others (Kruskal-Wallis test, P < 0.05). There were also significant differences in the expression of cFLIP among these three groups (Kruskal-Wallis test, *P *< 0.01). P < 0.05) (Table 2).

**Table 2:** IHC results for IGFBP-5 and cFLIP

Group	n	IGFBP-5 (+ ~ +++)				cFLIP (+ ~ +++)			
			
		N	%	*P_1_	**P_2_	n	%	*P_1_	**P_2_
Normal tissue	28	20	71.44			6	21.43		
CIN I	37	8	72.73	1.0000	1.0000	4	36.37	0.4238	0.4238
CIN II/III	26	26	100.00	0.0045	0.0212	20	76.92	<0.0001	0.0275
Cancer tissue	40	18	45.00	0.0308	<0.0001	33	82.50	<0.0001	0.5778

* P < 0.05 vs. normal tissue, ** P < 0.05 vs. adjacent abnormal tissue

### The relationship between IGFBP-5 and cFLIP expression and clinicopathological parameters

There were significant differences in IGFBP-5 protein expression among CIN stage I, II, and III samples. In CC samples, the degree of positive staining was related to clinicopathological stage, lymph node metastasis, and the degree of cell differentiation (P < 0.05). There were also significant differences in the level of cFLIP expression among the CIN stage I, II, and III groups (P < 0.05), and this expression level was related to pathological differentiation in CC (P < 0.05) (Table 3). Correlation studies were carried out using the Spearman and Kendall tests.

**Table 3:** The relationship between expression of IGFBP-5 and cFLIP and clinicopathological parameters in CC

clinical parameter	n	IGFBP5				n				cFLIP
		
		-	+	%	P		-	+	%	P
Lymph node metastasis										
existence	12	10	2	16.67		12	2	10	83.33	
Nonexistence	28	12	16	57.14	P < 0.05	28	5	23	82.14	P > 0.05
Clinical stage										
Stage I	26	11	15	57.69		26	6	20	72.92	
Stage II	14	11	3	21.43	P < 0.05	14	1	13	92.86	P > 0.05
Pathological differentiation										
well differentiated	24	6	18	75.00		24	7	17	70.83	
moderately or poorly differentiated	16	12	4	25.00	P < 0.05	16	0	16	100.0	P < 0.05

P values represent multiple comparisons within groups

### PCR results

**Figure 1:**
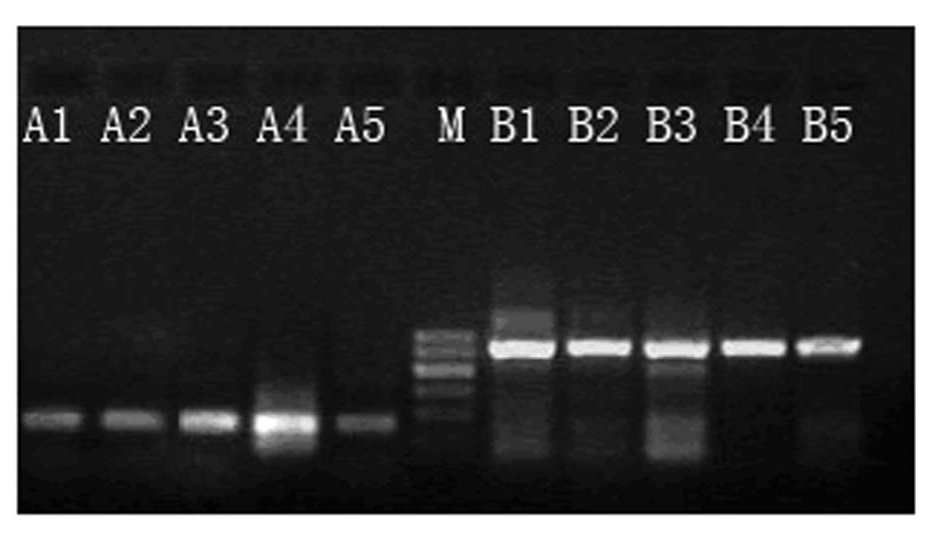
**Expression of IGFBP-5 (154 bp, A-lanes) and β-actin (540 bp, B-lanes) mRNA**. M = Marker, A1 = Normal cervical tissues group, A2-5 respectively express CIN I, II, III, and cervical squamous cell carcinoma groups.

**Figure 2:**
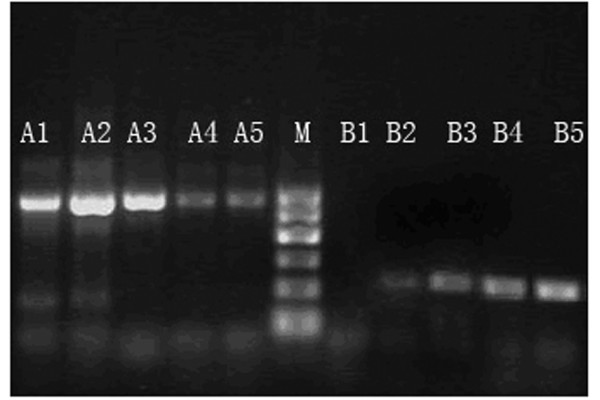
**Expression of cFLIP (226 bp, B-lanes) and β-actin (540 bp, A-lanes) mRNA**. M = Marker, B_1 _= Normal cervical tissues group, B_2–5 _respectively express CIN I, II, III and cervical squamous cell carcinoma groups.

**Figure 3:**
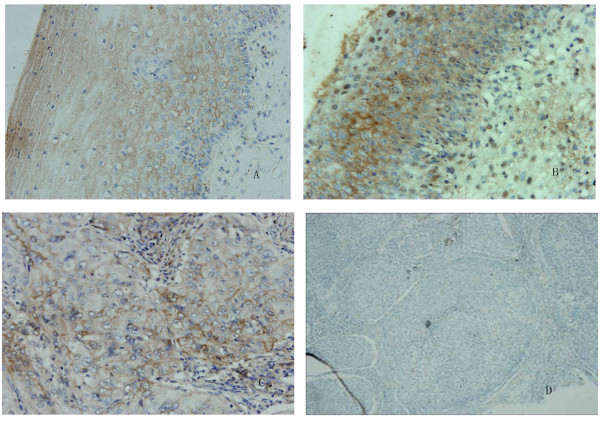
**Immunohistochemical detection of IGFBP-5 and cFLIP in patient tissues**. A, Expression of IGFBP-5 in CIN I tissue: ++(×400); B, Expression of IGFBP-5 in CIN II tissue: +++ (×400); C, Expression of cFLIP in cervical cancer tissue: ++ (×400). D, Expression of IGFBP-5 in cervical cancer tissue: - (×200).

The intensity (gray level) ratios of IGFBP-5/β-actin and cFLIP/β-actin were determined so as to represent the expression levels of IGFBP-5 and cFLIP mRNA. Larger ratios correlated with higher levels of expression of the target gene. Expression of IGFBP-5 were highest in the CIN stage II and III groups (1.0500 ± 0.0875), which were 4.94-fold higher than the relative expression levels of the normal group (0.2124 ± 0.0795) and 2.92-fold higher than those of the CC group (0.3600 ± 0.0575). The expression level in the CC group was in turn significantly higher than that of the normal group (P < 0.05) (Fig. 1). The highest expression of cFLIP mRNA was observed in the CC group (6.8874 ± 0.6663), which was 2.26-fold higher than that of the CIN stage II and III groups (3.0426 ± 0.0819). The lowest expression level was detected in the normal group (0.0246 ± 0.0100; P < 0.05) (Fig. 2 and Fig. 3).

## Discussion

Insulin-like growth factor (IGF) -I and IGF-II are important somatomedins in humans. Rather than moving freely through the blood and tissue fluids, these proteins bind to IGFBPs, mainly IGFBPs 1–6. IGFBPs inhibit the activity of IGF by tightly adhering to the ligand, though some binding proteins also activate the insulin-like growth factor [1]. Therefore, IGFBPs have recently received more recognition as potential tumor suppressors in the occurrence and development of tumors.

IGFBP-5 can inhibit the proliferation of some tumor cells. It has been reported that the down-regulation of IGFBP-5 correlates with the formation of oral keratinocyte cell tumors and IGFBP-5 over-expression in renal granular-cell tumor and fibroblast cell lines [2]. In breast cancer, IGFBP-5 interacts with various extracellular matrix components, and it is regulated by proteolysis and hormones that act on the mammary gland [3]. Pell et al. reported insulin-independent spontaneous anti-apoptosis activity of IGFBP-5 during the course of myogenesis [4]. Another study also showed that an IGF-independent mechanism could mediate the effect of IGFBP-5 on osteoprogenitor cells [5]. IGFBP-5 was also shown to enhance growth inhibition induced by tumor necrosis factor (TNF)-α. In cancer cells, IGFBP-5 activated the caspase-8 signal transduction pathway, increased the structure sensitivity to TNF-α, and induced the internal apoptosis pathway [6].

According to the results of the present study, with increasing severity of CIN, the expression of IGFBP-5 increased at both the mRNA and protein levels. We presume that in intraepithelial neoplasia, the body compensatorily up-regulates the expression of IGFBP-5, which activates the caspase-8 signal transduction pathway, increases the structure sensitivity to TNF-α, induces the internal apoptosis pathway, and delays tumor advancement. However, the expression of IGFBP-5 in the CC group was significantly lower than that of the CIN and normal cervical mucosa groups (P < 0.05). This trend was associated with clinicopathological stage, lymph node metastasis, and the degree of cell differentiation such that greater tumor differentiation and later clinical stages of CC were linked to lower levels of IGFBP-5 expression. The reason for this IGFBP down-regulation in CC remains unclear, though it may be explained by the down-regulation of HPV encoded proteins or the transcription of IGFBP-5 mRNA.

Irmler et al. [7] were the first group to find that cFLIP contains a death effect domain (DED), which blocks the death receptor pathway and inhibits apoptosis. The anti-apoptosis effect of cFLIP has been attributed to block the formation of death-inducing signaling complexes (DISC), the activation of caspases-8 and 10 and the course of the general caspase cascade. These effects are mediated by the two DEDs in the N-terminus of cFLIP that competitively bind to FADD and/or caspases-8 and 10. Under physiological conditions, cFLIP may protect normal cells from apoptosis induced by TRAIL. However, in tumor cells, over-expression of cFLIP inhibited the activation of the caspase-8 signal transduction pathway and cell apoptosis [8]. In traumatic brain injury, diverse mechanisms of cFLIP regulation could impact the degree of cell mortality and later programmed cell death [9]. A study demonstrated that cFLIP expression was also related to high-risk HPV infection and integration [10-12]. In this study, we found that the expression of cFLIP was significantly higher in the CC group than in the normal and CIN groups.

Our results suggest that in CC, decreased expression of IGFBP-5 might lead intracellular caspase-8 to not be effectively activated. Increased expression of cFLIP may cause the caspase-8 signal transduction pathway to be inhibited and stop the cascade reaction such that apoptosis of CC cells would be inhibited. This effect may block apoptosis signal transduction pathways induced by Fas, TRAIL, etc. and in turn promote the proliferation of tumor cells.

In this study, high-risk HPV was also detected. The rate of HPV infection was significantly greater in the CIN group than in the healthy control group (P < 0.05), though no differences were seen between the CIN and CC groups (P > 0.05). We also screened the hyper lesion of the cervix correlated with detection of HPV and found that the omission diagnostic rate was very low.

## Conclusion

In summary, IGFBP-5 was highly expressed in CIN, and it may participate as a tumor suppressor in the occurrence and development of cervical lesions. Down-regulation of IGFBP-5 expression was closely related to CC infiltration, metastasis, and differentiation, whereas cFLIP was highly expressed in CC. The interaction of these two effects may promote the progression of CC. Further study is required to confirm the roles played by these two proteins in the development of these diseases. Analysis of IGFBP-5 and cFLIP expression levels, may be useful tools for clinical diagnosis and differential diagnosis of CIN and cervical cancer.

## Competing interests

The authors declare that they have no competing interests.

## Authors' contributions

XJH: study design, data analysis, experimental studies, manuscript review. YZZ: the guarantor of integrity of the entire study, study design, experimental studies, data analysis, manuscript preparation. XL: clinical studies, manuscript review. LHM: experimental studies. YBQ: study design, manuscript editing.
